# Barriers and facilitators to hepatitis C (HCV) screening and treatment—a description of prisoners’ perspective

**DOI:** 10.1186/s12954-018-0269-z

**Published:** 2018-12-11

**Authors:** Des Crowley, Marie Claire Van Hout, John S. Lambert, Enda Kelly, Carol Murphy, Walter Cullen

**Affiliations:** 1 Irish College of General Practitioners Dublin, Dublin, Ireland; 2 School of Medicine University College, Dublin, Ireland; 3 Mater Misericordiae University Hospital, Dublin, Ireland; 4 Public Health Institute, Liverpool John Moores University, Liverpool, UK; 5 Irish Prison Service, Longford, Ireland

## Abstract

**Background:**

Hepatitis C virus (HCV) infection is a global epidemic with an estimated 71 million people infected worldwide. People who inject drugs (PWID) are overrepresented in prison populations globally and have higher levels of HCV infection than the general population. Despite increased access to primary health care while in prison, many HCV infected prisoners do not engage with screening or treatment. With recent advances in treatment regimes, HCV in now a curable and preventable disease and prisons provide an ideal opportunity to engage this hard to reach population.

**Aim:**

To identify barriers and enablers to HCV screening and treatment in prisons.

**Methods:**

A qualitative study of four prisoner focus groups (*n* = 46) conducted at two prison settings in Dublin, Ireland.

**Results:**

The following barriers to HCV screening and treatment were identified: lack of knowledge, concerns regarding confidentiality and stigma experienced and inconsistent and delayed access to prison health services. Enablers identified included; access to health care, opt-out screening at committal, peer support, and stability of prison life which removed many of the competing priorities associated with life on the outside. Unique blocks and enablers to HCV treatment reported were fear of treatment and having a liver biopsy, the requirement to go to hospital and in-reach hepatology services and fibroscanning.

**Conclusion:**

The many barriers and enablers to HCV screening and treatment reported by Irish prisoners will inform both national and international public health HCV elimination strategies. Incarceration provides a unique opportunity to upscale HCV treatment and linkage to the community would support effectiveness.

## Introduction

Hepatitis C infection (HCV) is a major global epidemic, with an estimated 71 million people chronically infected worldwide [1] . HCV carries a significant global disease burden with an estimated 399,000 dying annually from HCV-related liver failure and cancer [1, 2]. Unsafe injecting drug use (IDU) is the main route of HCV transmission in developed countries, with an estimated 20 million people who inject drugs (PWID) infected worldwide [2].

There are an estimated 10 million people incarcerated globally on any 1 day with many more coming in contact with the criminal justice system annually [2]. PWID are overrepresented in prison populations due to the criminalisation of drug use and the engagement in criminal activity to fund illicit drug habits [3, 4]. A number of HCV transmissions risks in prisoners have already been reported in the literature these include unsafe IDU, sharing of drug-taking paraphernalia, prison tattooing, and factors independent of these but linked to incarceration such as violent assault, sharing of tooth brushes and razors and possibly other unidentified factors [3, 5–7]. There is also evidence in the literature of increased HCV transmission among HIV-infected men who have sex with men (MSM), which is a concern in prisons without access to condoms [8]. The incidence of HCV in general prison populations is estimated at 1.4 per 100 person years (py), increasing to 16.4 per 100 py in inmates who inject drugs [3]. The global HCV prevalence in incarcerated populations is estimated at 26%, increasing to 64% among those with a history of IDU [3].

Prisoners have complex physical and psychological needs with poor access to and uptake of health services [9, 10]. While incarcerated, prisoners have better access to primary health care and lower mortality than when released back into the community [9, 11]. Prison provides structured routine, access to good nutrition and exercise, and removes many of the stressors experienced in the community [9]. HCV treatment can be effectively provided in prisons with outcomes equal or better than community-based treatment [12, 13]. Despite the recognised potential to screen and treat this high-risk group for HCV infection, uptake remains low [14].

The rate of imprisonment in Ireland is approximately 79 per 100,000 of population [15]. There are 3674 persons incarcerated in Ireland on any given day, and the annual turnover of prisoners is 14,182 [15]. In common with other prison populations, the majority of inmates are serving sentences of less than 12 months [15]. Similar to other prison populations globally, there are high levels of poverty, social deprivation, homelessness, early school dropout, unemployment, illiteracy, mental illness and drug use [4, 6, 9]. Over half of Irish prisoners report a history of opiate use with 43% reporting a history of injecting [6]. A 2000 study estimated the prevalence of HCV infection in the Irish prison population at 37% increasing to 81% % in those with a history of IDU [16]. A later study found a reduced HCV prevalence of 13% [6]. The Irish National HCV strategy (2014) identifies prisons as key locations to screen and treat HCV infection [17]. The Irish Prison Services (IPS) delivers primary health care through a network of general practitioners (GPs) and nurses in all 15 prison locations in the republic of Ireland (ROI). GPs and GP addiction specialist oversee blood-borne virus (BBV) screening. Presently in Ireland there is no structured approach to HCV screening in prisons. National guidelines recommend that all prisoners should be tested for HCV infection [18]; however, no data is available on screening and treatment uptake in Irish prisons. In-reach specialist hepatology services and fibroscanning services are provided in three prison locations in the Dublin area. These services provide HCV treatment equivalent to that provided in the community [19]. Direct acting anti-viral (DAA) therapies can only be prescribed by specialists, and their costs are funded by the National Health Service Executive (HSE) and do not impact on the prison health care budget.

Barriers to HCV screening and treatment in the community have been previously identified [20–22]. These include lack of knowledge and awareness of HCV, substance misuse, mental illness, poor motivation, fear of treatment, fear of liver biopsy, competing priorities, rigid hospital appointment system, treatment eligibility criteria and access, health insurance and transportation [20, 22, 23]. Similar blocks have been identified among prisoners [24, 25] and other unique challenges have been described for prison populations. These include short prison sentence, inter-prison transfers, prison bureaucracy and the cost to prison health care budgets. Critical enablers to HCV screening and treatment have been identified and these include in-reach hepatology services, improved models of health care delivery, increasing prisoners’ awareness and understanding of HCV infection and treatment options, educating both operational and clinical staff and involvement of peer educators in increasing knowledge and reducing stigma [26, 27].

Similar to other developed countries, Ireland has a large cohort of untreated chronically infected HCV prisoners [6, 16]. New screening techniques (dried blood spot testing), liver disease screening tools (elastography) and drug therapies (DAA) have revolutionised HCV screening and treatment, both in the community and in prisons [28–31]. Many studies have identified barriers and enablers to community-based HCV screening and treatment but very little research has been published on prison populations. HCV is now considered a preventable and curable infection, but challenges remain to accessing those infected [11, 32]. Prisons offer a unique opportunity to overcome these challenges, and increasing prison-based screening and treatment is an essential public health strategy in tackling this global epidemic [33]. The aim of this study is to augment the existing scant published literature on blocks and enablers to prison HCV screening and treatment, and inform the Irish National HCV treatment Program on strategies to maximise HCV screening and treatment uptake in the Irish prison population.

### Methodology

The location of the study was The Mountjoy Campus in Dublin, Ireland and involved two of the three institutions. Mountjoy Prison is a closed, medium security prison for adult males with an operational capacity of 554. The Dochas Centre is a closed, medium security prison for adult females with an operational capacity of 105. Four focus groups took place during 2017. Ethical approval was granted by the Mater Hospital Ethics Committee as part of Seek and Treat component of The European Hep Care Project and ratified by the Irish Prison Services.

Participants were recruited at both sites by open invitation through posters and directly by custodial and healthcare staff. Focus groups were conducted onsite and in a room located in the medical section of each prison to ensure privacy. No inducements were offered for participation. On completion of the focus groups, all participants were offered an opportunity to link with a specialist nurse. All interested prisoners (*n* = 46) were given a patient information sheet and a consent form to sign. Following a review of the literature on the topic, completion of a scoping review and consultation with the research group and national experts in the area a focus group guide was finalised. This guideline included a series of open-ended questions covering the following areas: experience of community-based and prison-based HCV screening and treatment, barriers and enablers to uptake, challenges related to incarceration and release, inter-prison variations in health care delivery and role of security staff and peers in prison HCV management.

Focus groups were facilitated by an experienced team of facilitators. Researcher 1 (DC) and 3 (CM) facilitated focus group 1, 2 and 4 (*n* = 37). Researcher 2 (MVH) and researcher 3 (CM) facilitated focus group 3 (*n* = 9). The average time for the focus group was 75 min with a range between 45 and 90 min. The focus groups were recorded and the audio files were transcribed using Microsoft Word 10 by researcher 1(DC). A grounded theory approach informed both the collection and analysis of the data. QSR NVivo 10.0 was used for organising and thematic coding of the transcribed data. The thematic coding was revised with the analysis of each focus group, and analysis ceased when thematic saturation was achieved (agreed by researcher 1 and 2).

## Results

The following themes related to barriers to both HCV screening and treatment emerged from the analysis, lack of knowledge, concerns regarding confidentiality, fear of being stigmatised, inconsistent access to prison health services with delays in screening and receiving results. Further themes related to enablers were identified including access to health care, opt-out screening at committal, peer support, and stability of prison life which removed many of the competing priorities associated with life on the outside. Unique themes identified related to treatment included, fear of treatment and having a liver biopsy, the requirement to go to hospital and in-reach hepatology services and fibroscanning.

### Lack of knowledge

All focus groups identified lack of knowledge as a major block to engagement with HCV treatment services. Prisoners were aware of their own lack of knowledge and were often confused about the different types of hepatitis.

‘I didn’t know anything about it. I think I have (it) before I came in here but I don’t know if it was Hep B or C’. (Male aged 28 years)

‘You have a high risk of catching it even if you’re not using it so I don’t know if it was Hep C or Hep B ……., there should be more education on it’. (Female aged 30 years)

‘I wouldn’t have known anything about Hep C and we might be transferring it without knowing. Everyone should go and have a test done because you don’t have to be on drugs all the time for that to happen’. (Male aged 33 years)

‘I didn’t know about the Hep till I came to Dublin a year ago and I heard ‘be careful about the Hep’ and I was like what the fuck is Hep’. (Female aged 24 years)

Many prisoners were confused about modes of transmission.

‘Think you can get it from smoking a rollie or from toilets. Haven’t got any information‘. (Male aged 28 years)

‘Does it live for six months on the floor?’ (Male aged 22 years)

Others commented on the misinformation that existed among other prisoners.

‘The misinformation that’s out there. People think once it’s there it’s there for life like you’re riddled’. (Male aged 22 years)

‘Most people who don’t have an understanding. You talk to someone who says something then you ask someone else and they’d tell you a completely different story. So, there’s misinformation. We still don’t know anything about it you know’. (Female aged 24 years)

### Fear of liver biopsy and treatment

Many prisoners spoke about their fear of treatment and the ‘horror’ stories they had heard from other inmates. Many found that their own experiences of treatment were much more positive,

‘Yeah, I heard of people losing their hair. It’d put you off. But it didn’t affect me one bit the way I thought it would. Need more education on that part of it’. (Male aged 28 years)

‘They used to get the injection but it made her sick or something. It did. She said she was very sick. Some people say I’d rather die than do the treatment’. (Female aged 26 years)

A number of prisoners described the fear of liver biopsy and similar to their experience found the reality of having the biopsy less onerous.

‘I remember years ago they’d go in through the top of the shoulder. So, I wouldn’t do it. Hear people ‘don’t get that liver biopsy’ but I went through it myself.... it was grand, no pain’. (Male aged 34 years)

‘‘They’re going to hit your lung’ just scaremongering’. (Male aged 27 years)

### Concerns regarding confidentiality

Prisoners expressed concerns regarding confidentiality. Some believed that non-medical staff had access to their medical records.

‘They (prison officers) say they won’t check but that’s bullshit you can go into any computer and access whatever you want’. (Male aged 28 years)

Many explained that the process of being called for bloods and hospital appointments was not confidential and prisoners were often called on the landing for certain blood tests and hospital appointments which revealed their medical status to the other prisoners and security staff.

‘Getting called for tests as you walk onto the landing and there, you’re getting called for blood test and people see you going and say why’s he getting a blood test, why why why you know?’ (Male aged 34 years)

### Fear of being stigmatised

Coupled with anxiety around confidentiality was the fear of being stigmatised by other prisoners and staff if they became aware of their HCV status.

‘There’s a stigma attached to it. There is!!’ (Female aged 25 years)

‘Then one of them called me aside because it’s gone around that I had the virus. That’s why I got sacked out of the kitchen because I had the virus.... Pure ignorance to take me out of the kitchen, just because I had Hepatitis C and at that stage it was gone’. (Male aged 36 years)

‘Yeah definitely! They feel like they’re gonna get judge’. (Female aged 26 years)

Many prisoners described a double stigma, the first associated with their HCV status and the second with being identified as a prisoner in a hospital setting. The policy of handcuffing male prisoners for security reason while attending out-patient appointments was identified as increasing the chances of experiencing stigma and shame. There appeared to be regional variation, with prisoner have more negative experiences in rural areas.

‘When I was in the Joy (Mountjoy Prison) you’d hate it because you’d have chains all over you. Handcuffs and that shit’. (Male aged 26 years)

‘In Dublin when you’re brought to the hospital they’re more used to it. When you go into the hospitals in the country in handcuffs even the doctors and nurses are looking at you’. (Male aged 34 years)

‘Talking to me like a piece of shit, and this man was taking my bloods. It makes an awful lot of difference’. (Male aged 36 years)

### Systemic barriers

Many participants expressed frustration at the many systemic blocks to HCV screening and treatment they experienced while incarcerated. These included delays in having bloods taken

‘Could be months down the line and they forget about it and then they come out of nowhere’. (Male aged 29 years)

‘Not chased up, it’s not efficient’. (Female aged 24 years)

Many also experienced long delays in receiving the results once the blood was taken.

‘I’ve waited two and a half years badgering for results so again that happens even if you did ask it takes too long so need to move quicker’. (Male aged 29 years)

‘They’re getting out before they even get blood tests. Then when you’re coming back in, they’re doing the same tests again and again’. (Male aged 36 years)

Others felt they had to wait too long for treatment despite being motivated and willing to engage with services.

‘Very slow process, to the point of treatment. For me it took years. For me I wanted the treatment as soon as possible and it still took ages’. (Male aged 36 years)

### Enablers

#### Opt-out screening at committal

Screening on comital was seen by most inmates as an enabler to treatment describing it as ‘more private’ and ‘more suitable’. Some proposed an opt-out type of screening program.

*‘*Make it automatic, straight away when you come in on committal’. (Male aged 26 years)

‘It should be done when you land in the prison. Should be just done’. (Female aged 44 years)

However, some participants were concerned that committal was already a stressful time for many inmates adapting to their new surroundings with some having to manage withdrawals.

‘A big group to do everyone there and then people are coming in with withdrawals ... it’s a difficult situation for us to be in too. That’s why they leave it for a while so people fit into the routine. Although if you’re only remanded for a week you wouldn’t get it’. (Male aged 34 years)

### In-reach hepatology

Participants identified the presence of in-reach hepatology services at both locations as a facilitator to engagement with HCV treatment. The availability of on-site specialist hepatology reduced the need for patients to attend hospital outpatients.

‘It’d be much better if the services were in the prison so you didn’t even need to go to hospital’. (Female aged 29 years)

### Access to in-reach fibroscanning

The majority of prisoner expressed satisfaction with access to and the experience of fibroscanning. They described it as ‘no problem’, ‘just a scan’ and ‘simple’. They identified it as an enabler to screening and treatment.

‘People go and get tested quicker’. (Male aged 34 years)

‘No hesitation at all, the woman that does it is nice. She explains it all to you, she’s good at talking simple’. (Male aged 26 years)

‘If people know it’s nothing big just a bit of gel then it’s no problem. When she showed me the machine it was no problem. Everyone would jump on that a lot quicker. Test so easy to get done’. (Male aged 28 years)

Participants highlighted that they had quicker and easier access to fibroscanning within prison than in the community.

‘It’s hard to get an appointment on the outside but it only takes 5-10 minutes in the (prison) clinic. When you know it’s that simple you’ll go 10 times quicker than hospital’. (Female aged 24 years)

‘Outside like…if you have an appointment you’d put it off and put it off. You’ve nothing but time in this place, It’s easier’. (Male aged 32 years)

### Stability of prison life eliminating competing priorities

All focus group participants agreed that prison afforded and ideal opportunity to engage with HCV screening and treatment. Prison eliminated many of the blocks experienced by this cohort in the community in particular, homelessness, personal motivation, competing priorities, access to health care and drug treatment.

‘I think you take the opportunity while you’re here instead of.... Especially when you’re in prison’. (Female aged 28 years)

‘You make excuses on the outside, make excuses about everything. In prison 100% you do it’. (Female aged 28 years)

‘I’m in jail now I better get sorted ... outside to get to the doctor, accommodation, drug use same kind of thing if you’re not getting accommodation you’re not going to go to any doctor. No accommodation and on drugs you don’t chase any of that up’. (Male aged 34 years)

‘Every single time I got out, I go to nothing. I said it to welfare, when I get out of here I have nowhere to go, I don’t want to get out and go to nothing. Walking around saying stuff and it’s like with hospital appointments or anything you don’t think about it’. (Male aged 38 years)

‘Hard enough for us to cope as it is outside with everyday life without throwing that on top. The opportunity to do it in prison you don’t have all the stresses of life to go with it, you’re more willing to take it on’. (Female aged 36 years)

‘Yeah going back out with no address you know... walking down the streets, you just wouldn’t go to hospital. Prison, quieter’. (Female aged 25 years)

### Peer support

Many participants identified peer educators as a potential facilitator to HCV screening and treatment. A number of prisoners had experienced mass HIV and TB screening programs involving Red Cross peer workers while serving previous sentences and described it as facilitating their engagement. They described trusting the peers, in particular those prisoners who had completed HCV treatment.

‘Someone who’s been through it and knows about it and knows about the body. Someone that’s been through it that’s been through the treatment that understands it’. (Male aged 28 years)

‘It is helpful when you’re talking to someone like that and they know what they’re talking about, it’s comforting’. (Male aged 34 years)

‘Yeah from a prisoner to a prisoner. It’s not like you’re going to be a teacher giving a lecture. You’re just sitting down talking about how you catch it and just educating people’. (Female aged 27 years)

‘Prisoners would give more time to other prisoners. You lose track with nurses because you just get fed up sometimes Where if it came from a prisoner who had it you’d listen more because you relate to what they’re after going through so at least you’d have a bit more understanding at the end of the day because you know they’re not judging you’. (Female aged 28 years)

## Discussion

Many of the barriers to HCV screening and treatment in prisoners identified in this study have been reported previously in earlier studies conducted in the pre-DAA era [24, 25]. As outlined in the introduction these include lack of knowledge and awareness of HCV, poor motivation, fear of treatment, liver biopsy and stigma, competing priorities and prison bureaucracy. Lack of information regarding HCV and its management and fear of treatment are recognised as challenges to HCV elimination in PWID and prisoners [20, 24]. Much of the fear surrounding treatment is related to interferon-based therapies and the historical requirement for pre-treatment liver biopsy [34]. Pan-genotypic DAA and non-invasive mobile elastography have simplified HCV treatment [35, 36]. The findings from this study, the first conducted on this issue in the DAA era, supports the need for a program of education to disseminate this information among PWID and prisoners, who still report fear as a barrier to engagement.

Participants identified peer educators as a facilitator to engagement with health services while incarcerated and important sources to access health information. The importance of peer to peer education is well documented [37]. Peer education has been adopted in health promotion in various settings because of its cost-effectiveness over professionally delivered services [38]. Furthermore, peers are seen by other prisoners as a credible source of information and have the potential to address the lack of HCV-related knowledge and stigma reported among prison populations [38].

Study participants experienced delays in accessing HCV screening and in receiving results. It is recognised that HCV screening programs in prisons are often ad hoc, inconsistent and incomplete [18, 39–41]. This research found an inconsistent approach to HCV screening with many prisoners only being tested at their own request. Consideration should be given to introducing an opt-out screening program on committal to prison in Ireland [18, 40, 41]. This screening strategy has been shown to be cost effective and has the potential to reduce HCV transmission and HCV-related liver disease primarily in the community [40, 42]. It was also supported by many of the focus group participants. Importantly, it has the potential to reduce stigma [41, 43]. There was widespread support for opt-out screening at committal from the participants. The routine and structured nature of the committal process was seen as a means to embed HCV screening as a routine part of prison health care. A small number of participants expressed concerns about adding screening into an already stressful time for new committals that might be struggling with withdrawal symptoms. This concern has been reported previously in the literature [40, 44].

Research shows prison-based HCV treatment to have equivalent or better outcomes to community and hospital-based treatment if the prisoner was not released or transferred during treatment [12, 31]. Despite their high cost, the use of DAAs in prison populations, are shown to be cost effective [45]. In Ireland, in-reach hepatology services exist in three institutions and two of these are included in this study. Prisoners identified these services as enablers to screening and treatment. These services reduce the need for hospital appointments, save on prison escorts, reduce risk to the general population and the embarrassment and stigma experienced by prisoners when attending these services while hand cuffed. In Ireland, the handcuffing of patients for hospital visits only occurs in the male prison population. In this study, the female prison focus group did not experience the same stigma and embarrassment as their male counterparts when attending for hospital appointments, with many enjoying ‘the day out’ as break from the monotony and boredom of prison life. Reviewing this policy may have an impact on compliance and uptake of HCV treatment.

Consideration should be given to piloting in an Irish setting other prison HCV treatment delivery models, shown to be effective in other jurisdictions. These include nurse-led clinics, teleconferencing and upskilling prison general practitioners and addiction doctors [46–48]. Different models may work best for different prisons depending on HCV prevalence, the structure and skill set of local health care teams and the availability and relationships with specialist hepatology services.

Any HCV screening and treatment model adopted by the IPS needs to take into consideration the need for continuity of treatment in the event of an inter-prison transfer or community release both identified as barriers to completing HCV treatment. Prisoners are often released without notice or pre-release planning. Linking community and prison in-reach hepatology will reduce the risk of patient drop-out on release. Inter-prison transfers need to be organised in a way that ensures prisoners on HCV treatment are only transferred to prisons where their treatment can be continued. Continuity of treatment is a key component to the cost-effectiveness of active case finding and treatment in prisons and transitioning back to the community is now considered a high risk period for HCV transmission in prisons [49]. Focus group participants described the negative impact that transition back to the community with homelessness, unemployment, drug user and other competing impacts can have on HCV treatment compliance. HCV treatment is seen as a relative need and often not the most pressing in PWID’s life in the community [50].

A consistent theme expressed in all the focus groups was the stigma and shame felt by many of the prisoners who were HCV infected or had a history of drug use. This is well recognised in the literature [20, 24, 51]. Repeated concerns were voiced in the focus groups around confidentiality. Many prisoners believed that prison officers had access to their computerised health records. Some prisoners identified that having bloods taken or seeing certain staff members linked with hepatology services identified them among their fellow prisoners as drug users. Prisoners described being publicly called on their landings for certain appointments which were clearly associated with being assessed or treated for HIV/HCV infection. Many HCV-infected patients are also in receipt of methadone maintenance treatment (MMT). The provision of MMT in both study locations is a large daily operational exercise making it impossible to protect the confidentiality of those attending the services. Maintaining absolute confidentiality is difficult in prison settings [52, 53]. Despite such limitations every effort should be made to ensure medical confidentiality by educating and training of both clinical and non-clinical staff on the issue and having appropriate information sheets for prisoners on how their medical records are stored and who has access to them.

All participants favoured peer worker involvement in HCV management in Irish prisons. Peer educators are often used in prison setting to deliver education and training programs [54]. The model has also been shown to be effective in increasing HCV screening and treatment in community settings [23, 29]. Research shows high levels of satisfaction among service users and staff in community-based drug treatment clinics with this role [55]. There is further evidence to suggest that engagement in HCV care may be facilitated by the influence of peers who completed treatment [56]. The ETHOS Study in Australia reported a very strong positive response to peer workers by staff and service users which lead to improved access to services, a more client-friendly treatment environment and increased support to service users with assessment and engagement with HCV treatment [56]. Involving peer educators helps to dispel many of the myths regarding HCV treatment. It is also a very effective vehicle to develop education programs around HCV infection and treatment options. Peers can also be an effective support system for patients on treatment particularly in prison settings where traditional family and community support structures are absent. Many of the focus group participants identified the presence of a peer support network as an enabler to HCV screening and treatment.

The strength of this study is that we were able to evaluate how different groups discussed HCV together and how they debated the merits and weaknesses of identified blocks including their own experiences. The use of the focus group methodology allowed for the engagement of large numbers of prisoners with limited use of prison staff. This is an important consideration for any research conducted in real-life prison settings with limited staff resources and competing priorities. The engagement of both male and female prisoners was identified as a strength and increased the generalisability of the findings both nationally and internationally. There are a number of limitations to this study including; participants may not have revealed their complete HCV narrative in the presences of others, researcher 1 was known to the male participants and the involvement of only two of the 15 prisons located in the ROI. Apart from age and gender other demographics on focus group participants were not collected. Knowledge of incarceration and drug use history along with HCV status and treatment history of the participants could have increased the interpretation and understanding of the focus group narratives. While the focus groups were conducted in only two locations, many of the participants had experience of other prisons and contributed these during the interviews. This may increase the generalisability of the findings to prisons outside of Dublin.

## Conclusion

Irish prisons are a key setting to identify and treat HCV infected PWID. This important public health strategy can only be achieved by the elimination of identified barriers to HCV screening and treatment in Irish Prisons. The availability of short-acting, tolerable and highly effective DAA can eliminate many of these barriers but effective education programs highlighting the benefits of these treatments are required. Expanding the provision of HCV treatment to non-specialist health services such as general practitioners, within the prison and community, has the potential to increase HCV treatment uptake and outcomes. Opt-out screening at committal with engagement of peer educators has the potential to increase engagement but to maximise treatment uptake it is imperative that pre-treatment assessment and treatment is offered as early as possible in the sentence to optimise completion and outcomes. The expansion of in-reach hepatology services and peer educators to all prisons in the ROI should be considered. This research identified the fear of stigma as a major barrier to engagement with HCV treatment. Efforts to upscale training and education among security and health care prison staff are required. At a broader policy level, consideration should be given to the de-criminalisation of drug users and the development of health services underpinned by inclusion and acceptance.

## Abbreviations

BBV: Blood borne virus
DAA: Direct acting anti-viral
GP: General practitioner
HCV: Hepatitis C virus
HIV: Human immunodeficiency virus
HSE: Health Service Executive
IDU: Injecting drug use
IPS: Irish Prison Service
MMT: Methadone maintenance treatment
PWID: People who inject drugs
PY: Person years
ROI: Republic of Ireland
